# Use of Cyrene™, as an alternative to dimethyl sulfoxide, as a diluent for Melatonin to determine its in vitro antimicrobial capacity

**DOI:** 10.1007/s00203-024-04151-9

**Published:** 2024-10-09

**Authors:** Ana Muñoz-Jurado, Francisco Jurado-Martos, Eduardo Agüera, Isaac Túnez, Begoña M. Escribano

**Affiliations:** 1 Department of Cell Biology, Physiology and Immunology, Faculty of Veterinary Medicine, University of Cordoba, Campus of Rabanales, Cordoba, 14071 Spain; 2 Maimonides Institute for Research in Biomedicine of Cordoba, (IMIBIC), Cordoba, 14004 Spain; 3 CICAP-Food Research Centre, Pozoblanco, Cordoba 14400 Spain; 4 Neurology Service, Reina Sofia University Hospital, Cordoba, 14004 Spain; 5 Department of Biochemistry and Molecular Biology, Faculty of Medicine and Nursing, University of Cordoba, Cordoba, 14004 Spain

**Keywords:** Bacterial growth, Hormone of darkness, Minimum inhibitory concentration, Solvent

## Abstract

Melatonin (MLT) is a methoxyindole that has potent antioxidant actions, anti-inflammatory, and antiapoptotic capacity. However, its in vitro antibacterial capacity has been the least studied of its properties. Dimethylsulfoxide (DMSO) has been the most used solvent for these tests, but it shows an antimicrobial effect if it is not dissolved. Cyrene™ is a new solvent that has emerged as an alternative to DMSO. Therefore, this study aimed to determine the antimicrobial capacity of MLT by MIC assays, using Cyrene™ as a solvent. Likewise, the solubility of MLT in this solvent and whether it exerted any effect on bacterial growth at different percentages was also determined. Different dilutions of MLT in Cyrene™ with different concentrations, were prepared. No growth inhibition caused by MLT was observed. The growth inhibition observed was because of Cyrene™. The maximum amount of MLT that can be diluted in 100% Cyrene is 10 mg/mL, but this percentage of solvent shows a bactericidal effect. Therefore, it must be dissolved at 5% to avoid this effect, so only 4 mg/mL of MLT can be diluted in it. Therefore, if no other solvents are available, the in vitro antibacterial role of MLT cannot be adequately assessed.

## Introduction

Melatonin (MLT) (N-acetyl-5-methoxytryptamine) is widely known for its physiological role as a sleep regulator (Reiter 1991; Escribano et al. 2014; Carrascal et al. 2018; He et al. 2021; Muñoz-Jurado et al. 2022a). However, it has a multitude of functions due to its antioxidant, anti-inflammatory, anti-apoptotic, oncostatic, free metal chelating and antibacterial properties (Tekbas et al. 2008; Kostoglou-Athanassiou 2013; Escribano et al. 2014; Galano et al. 2021). The in vitro antibacterial property of this hormone has been the least studied if we compare it with its other properties, and until now it has been related to its antioxidant and anti-inflammatory capacity or its ability to chelate heavy metals (aluminum, cadmium, copper, iron, lead and zinc) (Reiter et al. 2016), since bacteria are strongly dependent on them for their growth. Thus, it has been observed that the binding of MLT to iron (Castro Sánchez et al. 2014) causes a reduction in the cytoplasmic availability of this metal, which may inhibit bacterial growth (Castro Sánchez et al. 2014; Zhang et al. 2021). Also, in vivo studies have shown that MLT may be useful in combating sepsis and septic lesions due to its antioxidant and anti-inflammatory actions (Hu et al. 2017). In this way it has been seen that the exposure of patients to MLT reduces the risk of recurrent infection by *Clostridioides difficile* by 21.6% (Sutton et al. 2022). In this sense, studies carried out in mice infected with *Staphylococcus aureus* and *Escherichia coli* show that the exogenous administration of MLT produced an attenuation of symptoms through the modulation of proinflammatory cytokines and antioxidant enzymes (Bishayi et al. 2016).

On the other hand, in vitro studies that analyze the antibacterial capacity per se of MLT are limited and, in some cases, contradictory. While some studies confirm that MLT has an inhibitory effect on the growth of *Staphylococcus aureus* (Tekbas et al. 2008; Hatem 2011; Castro Sánchez et al. 2014) *Streptococcus mutans*,* Staphylococcus epidermidis*,* Escherichia coli* (Castro Sánchez et al. 2014), *Enterococcus faecalis* (Hatem 2011), *Pseudomonas aeruginosa* and *Acinetobacter baumannii* (Tekbas et al. 2008), *Porphyromonas gingivalis*, *Fusobacterium nucleatum* and *Aggregatibacter actinomycetemcomitans* (Ganganna et al. 2021). Others, on the contrary, did not observe an antibacterial effect of MLT against *Staphylococcus aureus*,* Pseudomonas aeruginosa*,* Proteus vulgaris*,* Aeromonas hydrophila*,* Bacillus subtilis* and *Escherichia coli* (Wang et al. 2001).

The cause of these contradictions in in vitro assays could be due to the fact that one of the limiting factors, when carrying out studies of bacterial susceptibility to MLT, is the low solubility of this molecule in water. Therefore, different solvents must be used to dilute it. Usually, ethanol or dimethyl sulfoxide (DMSO) have been used for this type of studies. However, it has been proven that MLT dissolved in ethanol precipitates (Tekbas et al. 2008). DMSO, on the other hand, shows an antimicrobial effect if it is not diluted, which is why the guidelines of both the European Committee on Antimicrobial Susceptibility Testing (EUCAST) and the Clinical and Laboratory Standards Institute (CLSI), suggest that concentrations of this solvent do not exceed 1% for this type of assay. This prevents dilution of high concentrations of MLT. This fact was proven in trials prior to this study, in which the maximum concentration of diluted MLT was 4 mg/mL in a 1:10 solution of DMSO: H_2_O. These concentrations of diluent and MLT do not allow us to affirm the possible antibacterial capacity of the hormone. In view of this, other authors’ claims that MLT, diluted in DMSO or ethanol, has antibacterial capacity in vitro are questionable, as it is impossible to test this quality with these solvents.

Recently, a new solvent called Cyrene™ (dihydrolevoglucosenone) has been developed, which has emerged as an alternative to DMSO. Cyrene is an aprotic dipolar solvent that is derived from waste biomass. Furthermore, Cyrene is a sustainable, non-toxic and low viscosity solvent, which is already successfully applied as an alternative solvent for different chemical reactions in the pharmaceutical industry (Grune et al. 2021). The evidence would suggest that the solubilizing power of Cyrene is comparable to that of DMSO (Camp et al. 2020). Likewise, Camp et al. (2020), claim that Cyrene can be an improvement over DMSO as a vehicle for antimicrobial drug discovery. However, Cyrene has not yet been tested as an MLT diluent, to evidence the possible in vitro antibacterial capacity of the hormone. Furthermore, it is not yet known exactly whether Cyrene could be toxic at a certain dose, nor the amount of MLT that can be diluted in it.

In view of the above, the main objective of this study was to test the in vitro antibacterial capacity of MLT using a new solvent, Cyrene ™. All of this will be carried out using minimum inhibitory concentration (MIC) assays. As secondary objectives, it was established: (1) to analyze the dissolution capacity of MLT in Cyrene; (2) to assess whether there are doses of Cyrene that could be toxic in bacterial growth assays. These secondary objectives will serve to determine the dose of Cyrene as an MLT diluent in MIC tests, of which there is no current evidence.

Two bacterial strains, representing the Gram-negative and Gram-positive groups, *Escherichia coli* (*E.coli*) and *Staphylococcus aureus* (*S.aureus*), respectively, have been used in all tests.

## Materials and methods

### Experimental protocol

To determine the antimicrobial effect of MLT, MIC was performed following the broth microdilution method, described by the Clinical & Laboratory Standards Institute (CLSI) (CLSI 2015).

**Fig. 1:**
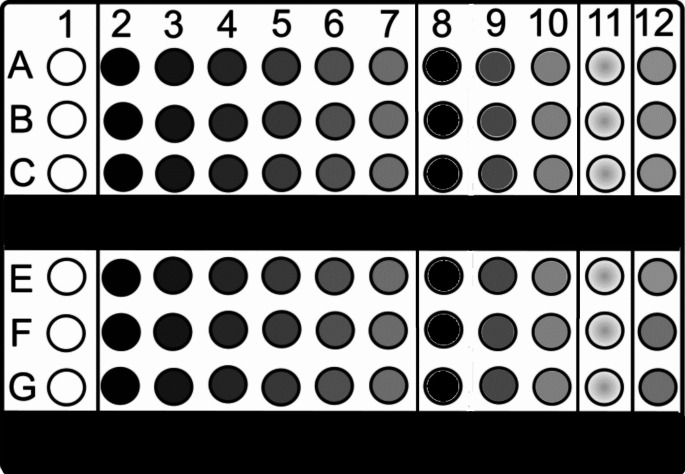
Microtiter plate layout. In rows A, B and C, the *Staphylococcus aureus* inoculum was tested. In rows E, F and G, the *Escherichia coli* inoculum was tested. Column 1 corresponds to the negative control, in which no bacterial inoculum is added, containing only 100 µl of Mueller Hinton Broth (MH) and 100 µl of the dilution of melatonin and Cyrene (MLT-Cyrene). Columns 2 to 7 correspond to the melatonin minimum inhibitory concentration (MIC) tests, performing serial twofold dilutions of MLT-Cyrene solution with MH medium. Columns 8 to 10 correspond to the tests of the effect on bacterial growth of Cyrene, diluting it with MH medium. Column 12 corresponds to the positive control, in which only MH and bacterial inoculum are added. In column 11, the effect of the control antibacterial agents was analyzed, using Penicillin G (10 µg) for *S.aureus* and Enrofloxacin (5 µg) for *E.coli*

The assays were performed in 96-well microtiter plates (Clinicord S.L.). Six serial twofold dilutions of MLT were made in Cyrene, at different concentrations, using Mueller Hinton (MH) broth. Likewise, to test the possible antibacterial effect that diluent could have, three serial twofold dilutions were carried out with different percentages of it, in MH broth, but without MLT. Penicillin G and Enrofloxacin were used as control antimicrobial agents. The concentration of each column was evaluated in triplicate for each bacteria (Fig. 1).

The plates were incubated for 24 h at 37 °C. Bacterial growth was indicated by the presence of turbidity and a pellet at the bottom of the well (Tekbas et al. 2008; CLSI 2015). A microbiological culture was performed on blood agar (Trypcase Soy agar + 5% Sheep Blood (TSS) (bioMérieux), in those cases where no growth was observed, to evaluate the bactericidal effect.

### Preparation of MLT dilution in Cyrene

In order to study the effect of MLT and Cyrene on bacterial growth, 5 assays were carried out using dilutions of MLT-Cyrene at different concentrations.

Cyrene (Cyrene™; Sigma Aldrich) was used as solvent to prepare the melatonin dilutions (LGC Ltd., Purity: 99.17). Likewise, in each of the trials, the antibacterial effect per se of Cyrene was also analyzed.

- MLT (4 mg/mL)-Cyrene stock solution assay.

The MLT concentration used in this trial was 4 mg/ml in Cyrene (100%). The range of MLT concentrations to be tested in this assay was from 1000 µg/mL to 31.25 µg/mL. The percentages of Cyrene, analyzed in columns 8, 9 and 10, were 25%, followed by 12.5% and 6.25% (Table 1).

- MLT (8 mg/mL)-Cyrene stock solution assay.

A dilution of 8 mg/mL of MLT-Cyrene was prepared. The MLT concentrations analyzed from columns 2 to 7 were from 2000 µg/mL to 62.5 µg/mL. The percentages of Cyrene evaluated in this trial, in columns 8, 9 and 10, replicated those existing in columns 2, 3 and 4, starting from 25 to 6.25% (Table 1).

- MLT (10 mg/mL)-Cyrene stock solution assay.

In this assay, the MLT concentration was increased by preparing a 10 mg/mL dilution of MLT-Cyrene. Thus, the MLT concentrations analyzed from column 2 to 7 ranged from 2500 µg/mL to 78.125 µg/mL. For its part, the range of percentages evaluated for Cyrene was from 25 to 6.25% (columns 8 to 10), thus replicating the percentages of the solvent in columns 2, 3 and 4 (Table 1).

- MLT (4 mg/mL)-Cyrene (10%) stock solution assay.

A dilution of 4 mg/mL of MLT was prepared in Cyrene diluted 1:10 in MiliQ distilled water. In this case, the MLT concentrations studied ranged from 1000 µg/mL to 31.25 µg/mL. The percentages of Cyrene tested, in columns 8, 9 and 10, were 2.5, 1.25 and 0.625% (Table 1).

- MLT (4 mg/mL)-Cyrene (5%) stock solution assay.

For this test, a dilution of 4 mg/mL of MLT in Cyrene diluted 0.5:10 in MiliQ distilled water was carried out. The MLT concentrations in this trial are the same as in the previous trial. However, the percentages of Cyrene studied in this case were 1.25, 0.625, 0.3125% in columns 8, 9 and 10, respectively (Table 1).

**Table 1:** Summary of concentrations of Melatonin (MLT) in µg/mL and Cyrene™ in percentage (%) per column, in each of the assays carried out in the study. Column 1: negative control; columns 2–7: serial twofold dilutions of MLT-Cyrene; columns 8–10: serial twofold dilutions of Cyrene without MLT; Column 11: antibiotic control; column 12: positive control

		Column 1	Column 2	Column 3	Column 4	Column 5	Column 6	Column 7	Column 8	Column 9	Column 10	Column 11	Column 12
Assay 1	MLT (µg/mL)	2000	1000	500	250	125	62.5	31.25	0	0	0	0	0
	Cyrene (%)	50	25	12.5	6.25	3.125	1.56	0.78	25	12.5	6.25	0	0
Assay 2	MLT (µg/mL)	4000	2000	1000	500	250	125	62.5	0	0	0	0	0
	Cyrene (%)	50	25	12.5	6.25	3.125	1.56	0.78	25	12.5	6.25	0	0
Assay 3	MLT (µg/mL)	5000	2500	1250	625	312.5	156.25	78.125	0	0	0	0	0
	Cyrene (%)	50	25	12.5	6.25	3.125	1.56	0.78	25	12.5	6.25	0	0
Assay 4	MLT (µg/mL)	2000	1000	500	250	125	62.5	31.25	0	0	0	0	0
	Cyrene (%)	5	2.5	1.25	0.625	0.3125	0.156	0.0781	2.5	1.25	0.625	0	0
Assay 5	MLT (µg/mL)	2000	1000	500	250	125	62.5	31.25	0	0	0	0	0
	Cyrene (%)	2.5	1.25	0.625	0.3125	0.156	0.0781	0.039	1.25	0.625	0.3125	0	0

### Microplate inoculation

As a final step in the preparation of the microtiter plates for each of the assays, bacterial inoculation was carried out. For this, two reference strains were selected *Escherichia coli* (*E. coli*) (ATCC 11775; NCTC 9001) and *Staphylococcus aureus* (*S. aureus*) (ATCC 6538; NCIMB 9518) from the Spanish Type Culture Collection (CECT), both of which are representative of the Gram-negative and Gram-positive groups of bacteria, respectively. The bacterial strains, stored at -80 °C, were transferred to tubes with Brain Heart Infusion (BHI) medium (Oxoid Ltd) and incubated for 24 h at 37 °C. Subsequently, two bacterial inocula were prepared with the isolated strains according to the 0.5 McFarland standards [approximately 1-2 × 10^8^ colony forming units (CFU)/mL] (Tekbas et al. 2008; CLSI 2015). Next, 100 µl of each inoculum were added to tubes with 9.9 ml of MH, thus obtaining a final inoculum of 10^6^ CFU/mL. From this, the wells of each one of the plates were inoculated homogeneously with 100 µl and with a concentration of 10^5^CFU/mL.

## Results

### MLT solubility in Cyrene

After carrying out the different tests, we verified that up to 10 mg of MLT can be diluted in 1mL of 100% Cyrene. If Cyrene is diluted to 10% or 5%, the maximum amount of MLT that can be diluted is 4 mg.

### MLT (4 mg/mL)-Cyrene stock solution assay

In this assay, no growth pellet is obtained in any column, except in column 7, where there is an MLT concentration of 31.25 µg/mL and a Cyrene percentage of 0.78. On the other hand, in the columns where only Cyrene was added, no pellet was observed (Table 2).

**Table 2:** Summary of the growth obtained for each of the microorganisms *Staphylococcus aureus* (*S. Aureus*) and *Escherichia coli* (*E. Coli*) by columns, in each of the assays performed. (-): no bacterial growth; (+): bacterial growth

		Column 1	Column 2	Column 3	Column 4	Column 5	Column 6	Column 7	Column 8	Column 9	Column 10	Column 11	Column 12
Assay 1	*S.aureus*	-	-	-	-	-	-	+	-	-	-	-	+
	*E.coli*	-	-	-	-	-	-	+	-	-	-	-	+
Assay 2	*S.aureus*	-	-	-	-	-	-	+	-	-	-	-	+
	*E.coli*	-	-	-	-	-	-	+	-	-	-	-	+
Assay 3	*S.aureus*	-	-	-	-	-	-	+	-	-	-	-	+
	*E.coli*	-	-	-	-	-	-	+	-	-	-	-	+
Assay 4	*S.aureus*	-	-	-	+	+	+	+	-	-	+	-	+
	*E.coli*	-	-	-	+	+	+	+	-	-	+	-	+
Assay 5	*S.aureus*	-	-	+	+	+	+	+	-	+	+	-	+
	*E.coli*	-	-	+	+	+	+	+	-	+	+	-	+

### MLT (8 mg/mL)-Cyrene stock solution assay

As in the previous assay, growth is only observed in column 7 which, in this case, contains 62.5 µg/mL MLT and 0.78% Cyrene. Again, no growth was observed in the columns where Cyrene was added without MLT (Table 2).

### MLT (10 mg/mL)-Cyrene stock solution assay

**Fig. 2:**
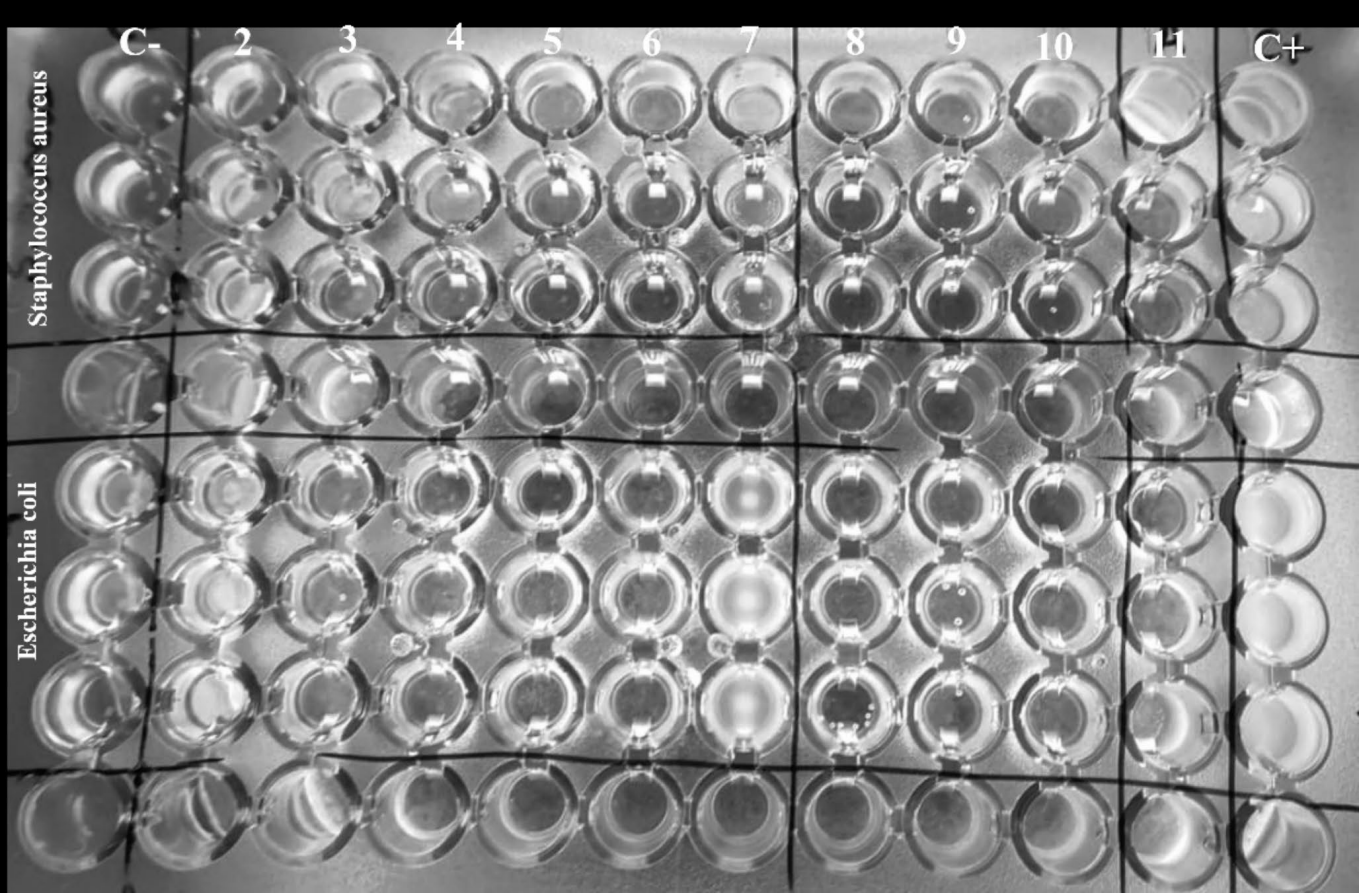
Microtiter plate made with 10 mg/mL melatonin-Cyrene solution. Rows A, B and C inoculated with *Staphylococcus aureus* and Rows E, F and G inoculated with *Escherichia coli*. Column 1: negative control; Column 11: Antibiotic control; Column 12: positive control. Columns 2–7: melatonin at concentrations of 2500, 1250, 625, 312.5, 156.25 y 78.125 µg/mL, respectively, in Cyrene (100%). Columns 8–10: Cyrene at a percentage of 25%, 12.5% and 6.25%, respectively

As in the two previously described assays, no growth is obtained in any of the wells, except for column 7. This contains an MLT concentration of 78.125 µg/mL and a Cyrene percentage of 0.78%. Likewise, no growth was observed in any of the wells containing only Cyrene (Table 2) (Fig. 2).

### MLT (4 mg/mL)-Cyrene (10%) stock solution assay

In this assay, no growth is obtained either in column 2 (1000 µg/mL of MLT and 2.5% Cyrene) or in column 3 (500 µg/mL of MLT and 1.25% Cyrene), the same behavior being observed for the two bacteria used. We also get no growth in columns 8 and 9, which only contain Cyrene at a percentage of 2.5 and 1.25%, respectively (Table 2).

### MLT (4 mg/mL)-Cyrene (5%) stock solution assay

No growth is observed in column 2, which contains 1000 µg/mL of MLT and 1.25% Cyrene, neither in the case of *S.aureus* nor in that of *E.coli*. For its part, we do not obtain growth in column 8, which contains Cyrene at 1.25%, but does not contain MLT (Table 2).

### Cyrene MIC and MBC

Because no possible bactericidal or bacteriostatic effects of MLT could be observed, but effects were observed by Cyrene, its bactericidal effect was analyzed. For this, a microbiological culture was carried out of wells of column 5 and 6, of trials 1, 2, and 3, which contain 3.125% and 1.56% of Cyrene, respectively. In the culture of column 5, no colony growth was obtained, while in contrast, in the culture of the wells of column 6, growth was obtained. Therefore, for these tests the MIC of Cyrene is set at 1.56% and the minimum bactericidal concentration (MBC) is set at 3.125%, for both microorganisms. Likewise, from the aforementioned assays, the wells of columns 8, 9 and 10, which did not contain MLT, were cultured and no growth was obtained after 24 h of incubation.

Cultures were also performed from the wells of columns 2, 3 and 8 and 9 of trial 4, and from the wells of columns 2 and 8 (1.25% Cyrene) of trial 5. In all of them, counts of more than 100 CFU/mL were obtained, so the bactericidal effect was ruled out for these percentages and a MIC of Cyrene of 1.25% was established for both bacteria.

With these results, the MBC/MIC ratio was calculated, for which values less than 4 were obtained. If the MBC/MIC ratio ≤ 4, the effect is considered bactericidal, but if the MBC/MIC ratio > 4, the effect is defined as bacteriostatic (Mogana et al. 2020). According to this and the MBC/MIC ratio obtained, we consider a bactericidal effect.

## Discussion

MLT is a very versatile molecule with a multitude of functions that are being widely studied. This indolamine is known to exhibit potent antioxidant actions, anti-inflammatory and immunomodulatory capacity and anti-apoptotic capacity, as well as an antitumor and neuroprotective effect (Reiter et al. 2000; Esposito and Cuzzocrea 2010; Rosales-Corral et al. 2012; Wang et al. 2013; Bahamonde et al. 2014; Tordjman et al. 2017; Muñoz-Jurado et al. 2022b). However, despite the large number of studies on melatonin, only a few analyze the in vitro antimicrobial activity of this hormone (Jasim et al. 2010), since the effects as an inhibitor of bacterial growth in vivo seem to be due to other properties of MLT.

To determine in vitro levels of susceptibility or resistance of specific bacterial strains to an antibacterial agent and to assess the activity of new agents on bacteria, the most widely used method is MIC (Wiegand et al. 2008; Kowalska-Krochmal and Dudek-Wicher 2021). This is the lowest concentration of an antimicrobial agent expressed in mg/L (µg/mL) that, under strictly controlled in vitro conditions, completely prevents visible growth of the strain (EUCAST 2000; Kowalska-Krochmal and Dudek-Wicher 2021). When carrying out a MIC assay with MLT, it is important to take into account that this molecule is insoluble in water. In recent years the development of ecological solvents is emerging, which is a key point of the so-called green chemistry (Camp 2018). An example of this is Cyrene, being a possible substitute for toxic dipolar aprotic solvents (Camp 2018), such as DMSO, produced in a simple synthesis from cellulose (Grune et al. 2021). Cyrene is becoming increasingly popular among green, non-mutagenic and non-toxic solvents, thanks to its biocompatibility and applicability (Citarella et al. 2022).

In the assays carried out, it was possible to prove, for the first time, that up to 10 mg MLT can be diluted in 1 mL of 100% Cyrene, which is a higher concentration than that achieved in assays prior to this study with DMSO (8 mg MLT in 1 mL of 100% DMSO). However, the need to dilute Cyrene, to avoid its antibacterial effect, means that only 4 mg of MLT can be diluted in Cyrene at either 10% or 5%, which is the same concentration that can be diluted in DMSO at 10%.

In assays 1, 2, 3, in which MLT concentrations of 4 mg/mL, 8 mg/mL and 10 mg/mL, respectively, diluted in 100% Cyrene, a complete inhibition of bacterial growth was obtained in almost the entire plate, including those wells in which Cyrene was added, without diluted MLT. Dilution of Cyrene to 10% and 5% (trials 4 and 5, respectively) also produced growth inhibition in the first columns of the plates of each assay. With these results, it can be established that the MIC of Cyrene, for both *S.aureus* and *E.coli*, is between 1.56 and 1.25%. The results obtained for Cyrene contradict those reported by Camp et al. (2020) in their study, in which they establish that the MIC_80_ values of Cyrene for *S. aureus* and *E. coli* are 8% and 5%, respectively, pointing out that Cyrene is not toxic to bacteria, despite the fact that in some of the analyzes carried out in their study, they use 100% Cyrene (Camp et al. 2020).

In none of the cases is an effect observed by the MLT, regardless of the concentration used, showing that the absence of bacterial growth obtained in the five assays carried out is due to the effect of Cyrene. Therefore, we cannot affirm that MLT has a direct effect on bacterial growth in in vitro assays as stated by Tekbas et al. (2008) in their study, in which they used a concentration of 2 mg/mL of MLT, dissolved in 100% DMSO, which is a lower concentration than the one used here and using DMSO at that percentage, which is known to affect bacterial growth. Our results also do not agree with those of Hatem et al. (2011), who use the same dilution of MLT in DMSO as Tekbas et al. (2008) (2 mg/mL in DMSO), nor with those of Castro-Sanchez et al. (2014), who used concentrations from 43 to 3µmol of MLT diluted in DMSO. Similarly, our results do not match those reported by Ganganna et al. (2021), who use a concentration of MLT in a range of 0.2 to 100 µg/mL, dissolved in ethanol. These studies concluded that MLT exerted an inhibitory effect on bacterial growth. However, the concentrations used in these studies are also lower than those used in the assays in this study (from 4 mg/mL to 10 mg/mL). Our results do agree with those reported by Wang et al. (2001), in which they obtained a lack of antibacterial activity of MLT, at a concentration of 200 µg/mL, on *Staphylococcus aureus*, *Pseudomonas aeruginosa*,* Proteus vulgaris*,* Aeromonas hydrophila*,* Bacillus subtilis* and *Escherichia coli.* In addition, Danilovich et al. (2022) report that the exogenous administration of MLT (15 or 150 mg/L) favored the growth and formation of biofilms in *Rhodococcus* spp. F27 and *Gordonia* spp. H19 (Danilovich et al. 2022).

Perhaps the lack of antimicrobial effect of MLT that we observed may be due to the ability of certain bacteria to produce this indolamine. Therefore, it may be necessary to administer a higher dose than that used here, which is above the physiological levels of MLT to which these microorganisms are exposed (He et al. 2021). This is confirmed by Wiid et al. (1999) in their in vitro study with *Mycobacterium tuberculosis*, in which they perform MIC trials, using MLT alone or combined with isoniazid. They report that MLT, in concentrations higher than those achieved physiologically, can by itself inhibit the growth of mycobacteria (Wiid et al. 1999). However, with the use of DMSO diluted 10-fold in distilled water as a solvent, it is not possible to prepare a dilution with MLT that exceeds 4 mg/mL. Cyrene allows higher concentrations of MLT to be diluted, reaching 10 mg of MLT in 1 mL of Cyrene. However, the bactericidal effect of this solvent implies the need to dilute it to at least 5% to reduce this effect. This means that the maximum dilution that can be made of MLT in diluted Cyrene is 4 mg/mL, as is the case with DMSO.

It is important to highlight that in in vivo studies with MLT, it has been shown to have a powerful effect against microbial infection in all cases, although not because of its antimicrobial activity, but because of other properties it has. An example of this is the study carried out by Carrillo-Vico et al. (2005), in which they found that MLT improves the survival of mice with septic shock through its pleiotropic functions as an immunomodulator, antioxidant, and antiapoptotic mediator (Carrillo-Vico et al. 2005). In the same way, Maestroni (1996), shows that indolamine exerts immunoregulatory effects through T-helper 2 (Th2) cell products (IL-4, IL-5…), which can modulate the secretion and/or action of inflammatory cytokines, which play an important role in the development of septic shock associated with endotoxemia. Furthermore, they report that a single injection of melatonin protects mice treated with a lethal dose of bacterial lipopolysaccharide (LPS) (Maestroni 1996). In this sense, studies from our research group conclude that MLT decreases intestinal dysbiosis in mice with experimental autoimmune encephalomyelitis (EAE), the animal model of multiple sclerosis, through the decrease of LPS and its binding protein (LBP) (Escribano et al. 2022). Likewise, Bishayi et al. (2016) demonstrate that melatonin administration during acute bacterial infection by *S.aureus* and *E. coli*, produced an increase in reduced glutathione (GSH) and superoxide dismutase (SOD) activity, with a concomitant decrease in lipid peroxidation and catalase activities, also reducing the levels of TNF-α (tumor necrosis factor-α), IFN-γ (interferon-γ), IL-6 (Interleukin-6), iNOS (inducible nitric oxide synthase), COX-2 (Cyclooxygenase-2) and CRP (C-reactive protein) (Bishayi et al. 2016). Also, the article published by Di et al. (2023), in which they evaluate the therapeutic potential of MLT administration to mice with septic myocardial injury, showing that pretreatment with MLT exerted a protective effect on sepsis and septic myocardial injury, which was related to the attenuation of inflammation and oxidative stress, improvement of mitochondrial function, regulation of endoplasmic reticulum stress (ERS) and activation of the AMPK signaling pathway (Di et al. 2023). Likewise, a recent article published by Shokri et al. (2024), shows that the use of Melatonin-loaded zinc and gallium nanoparticles for the treatment of bone infections, provide great potential as an antibacterial and osteogenic component in bone substitutes (Shokri et al. 2024). These in vivo investigations, together with the in vitro results of this study, could be decisive in deducing that MLT does not have an antibacterial effect per se, but rather acts on the cellular and molecular pathways that are activated when infection occurs in the body. Therefore, in order to determine whether MLT really has antibacterial capacity per se in in vitro tests, it is necessary to carry out research on other potential solvents, currently non-existent, that allow higher quantities of MLT to be diluted. A concentration of 4 mg/mL of MLT does not affect the growth of the strains of *S. aureus* and *E. coli* used in this study.

## Conclusions

The main conclusions that we can draw from this study are: (1) In 100% Cyrene, amounts of MLT of 10 mg/mL can be diluted. However, this concentration of Cyrene is toxic for the bacterial growth of the two strains used in this study; (2) A MIC for Cyrene is established between 1.56 and 1.25% for both microorganisms; (3) At concentrations of 5% and 10% Cyrene, the maximum concentration of MLT that can be diluted is 4 mg/mL; (4) There is no antibacterial effect in vitro by MLT at a maximum concentration of 4 mg/mL for the strains of *S. aureus* and *E. coli* used; (5) As long as the pharmaceutical industry does not develop solvents that do not affect bacterial growth, to make higher dilutions of MLT, this hormone has no in vitro antibacterial capacity.

## Data Availability

No datasets were generated or analysed during the current study.
